# Point Tenderness for 35 Years: A Rare Presentation of a Glomus Tumor in the Proximal Upper Extremity

**DOI:** 10.7759/cureus.60917

**Published:** 2024-05-23

**Authors:** Kavya Penmethsa, Aishwarya Kunta, Hannah Patel, Brandy M Bodiford

**Affiliations:** 1 Medical School, Alabama College of Osteopathic Medicine, Dothan, USA; 2 Family Medicine, Alabama College of Osteopathic Medicine, Dothan, USA

**Keywords:** cutaneous hyperesthesia, smooth-muscle actin, upper extremity pain, tenderness, glomus body, extradigital glomus tumor, glomus tumor, glomus tumor unusual location

## Abstract

Glomus tumors are rare benign neoplasms that are commonly found on the fingers and distal extremities. Clinically, they are often associated with a symptom triad of moderate pain, cold sensitivity, and point tenderness. These tumors are often not considered during a clinical workup due to their rarity and can be misdiagnosed due to their diverse clinical presentations.

Glomus tumors are made up of mesenchymal cells derived from glomus bodies, which are specialized arteriovenous (AV) anastomoses primarily responsible for thermoregulation. Microscopically, they present as intricate nests of endothelial cells surrounding glomus bodies, which can clinically manifest as point tenderness. Glomus tumors are usually benign and are commonly found in locations with a high concentration of glomus bodies such as the fingers. Extradigital tumors are very rare and usually not considered in primary diagnosis. This can lead to patients experiencing years and, in this case, decades of unexplained pain. The diagnostic workup for glomus tumors should include an initial Doppler ultrasound and a definitive diagnosis via immunohistochemistry (IHC). They can be completely cured with surgical excision. Although most glomus tumors are benign and easily treatable, they are often not considered in differential diagnoses when assessing for point tenderness.

This case illustrates an atypical presentation of a glomus tumor that caused 35 years of chronic pain and was incidentally misdiagnosed on imaging, leading to treatment delay by an additional eight months. This exemplifies the necessity of including glomus tumors within the differential diagnosis and diagnostic workup for point tenderness and soft tissue masses of the upper extremity.

## Introduction

Glomus bodies are arteriovenous (AV) anastomoses found throughout the epidermis that regulate body temperature through AV blood shunting mechanisms [1]. Glomus tumors consist of benign, modified smooth muscle cells similar to those found in a normal glomus body, and they represent less than 2% of soft tissue tumors [2,3]. They are typically found in regions that have an abundance of glomus bodies such as the dermis of the foot, palm of the hand, and subungual areas of the digits. However, there have also been documented cases of glomus tumors in visceral tissue such as the stomach, liver, and genitourinary tract [4]. They commonly occur in patients between the ages of 30 and 50 with a classic clinical triad of moderate pain, cold sensitivity, and point tenderness [2]. Most glomus tumors are less than 1 cm in diameter, making them difficult to palpate on physical exam [2]. Their prevalence is unknown as they have variable presentations on imaging, leading to common misdiagnoses such as venous malformations or hemangiomas [3]. This can lead to patients experiencing unnecessary chronic pain as emphasized by this case, illustrating a crucial need to incorporate glomus tumors in the differential diagnosis of point tenderness in the upper extremity.

This article was previously presented as a poster presentation at the 2024 Medical Association of the State of Alabama Annual Session on April 12, 2024.

## Case presentation

A 65-year-old male with a past medical history of hypertension, nephrolithiasis, gastroesophageal reflux disease (GERD), hyperlipidemia, lumbar radiculopathy, lumbar degenerative disc disease, and tobacco use presented for a routine follow-up visit complaining of worsening left upper extremity pain. The patient stated that he had been experiencing point tenderness and hyperesthesia in the left upper deltoid for the past 35 years. His symptoms began after a sweat gland excision procedure where a metal tourniquet was placed on his left arm in the same location of his pain. He stated that the pain and sensitivity only escalated since this procedure. Within the last two months, the pain in his left upper extremity began worsening, which he attributed to his young son regularly holding his arm and irritating the site. He stated that treating the area with Blue-Emu analgesic cream and Biofreeze did not improve his symptoms. He was prescribed gabapentin for lumbar radiculopathy and was told that it could potentially alleviate his upper extremity symptoms, but it was ineffective. The patient reported that he saw a dermatologist and had an electrolysis procedure done to the area with no pain relief. On physical exam, hyperesthesia and tenderness to light palpation were noted in a 2-cm area overlying his left deltoid. He denied associated rash, erythema, or previous trauma to the area. The patient was then prescribed lidocaine patches to assist in pain relief. 

**Figure 1:**
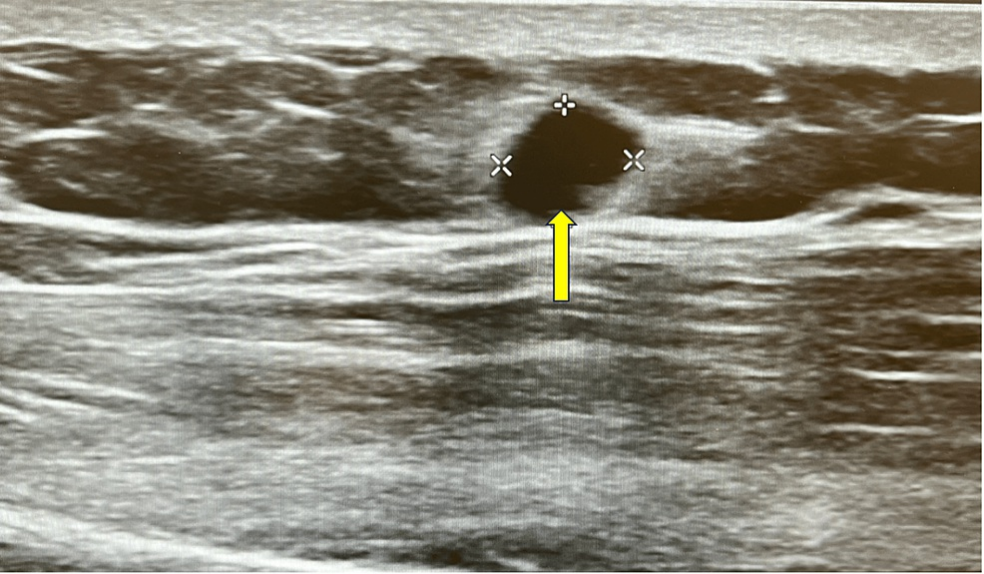
B-mode or gray-scale ultrasound image of the left proximal arm shows a 5×5-mm hypoechoic mass. The yellow arrow highlights the hypoechoic mass found on the gray-scale ultrasound.

**Figure 2:**
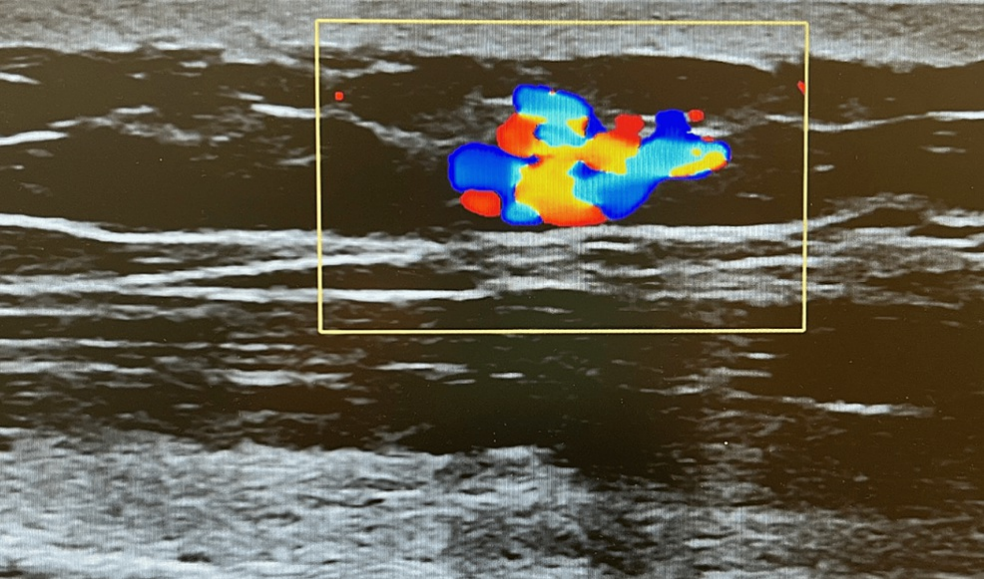
Doppler ultrasonography of the mass shows mixed AV blood flow, leading to a radiologic interpretation of AV fistula due to traumatic sequelae. The yellow box highlights the mixed blood flow seen on Doppler ultrasonography. AV: arteriovenous

Over the next two months, the patient was seen by various specialists for unrelated medical conditions and underwent duplex ultrasound of the left upper extremity (Figure 1). The radiology report endorsed an incidental finding of a 5-mm benign left upper extremity AV fistula most likely due to traumatic sequelae (Figure 2). At a follow-up visit eight months after the ultrasound, the patient reported continued worsening pain in his left upper extremity. He reported pain at rest and exacerbation of pain with increased emotional excitement and stopped riding his bike because the heat of the sun also caused him discomfort. He was prescribed oxycodone as needed for his lumbar degenerative disc disease and reported alleviation of his left upper extremity symptoms. The patient's clinical presentation coupled with the report of an AV fistula on imaging prompted a referral to general surgery. 

**Figure 3:**
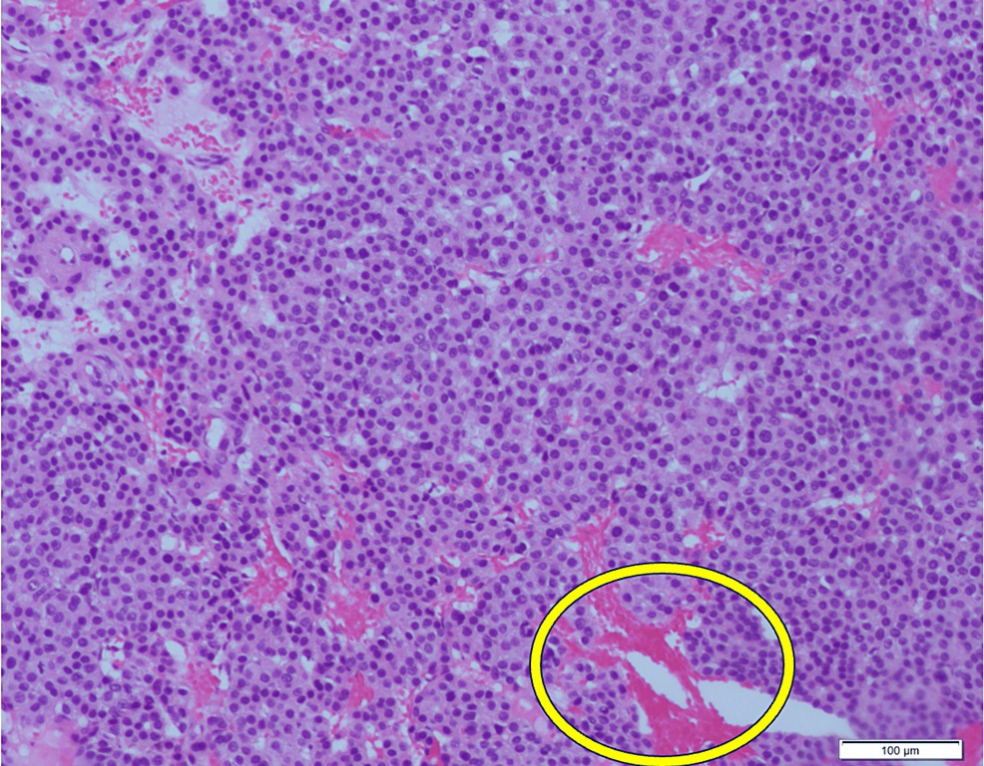
Microscopic image of excised left lateral deltoid mass measuring 0.7×0.6×0.5 cm at 10× magnification with H&E staining shows proliferation of round, bland cells in a background of eosinophilic cytoplasm and thin-walled blood vessels interspersed throughout. The yellow circle demarcates a blood vessel in a background of glomus tumor cells. H&E: hematoxylin and eosin

**Figure 4:**
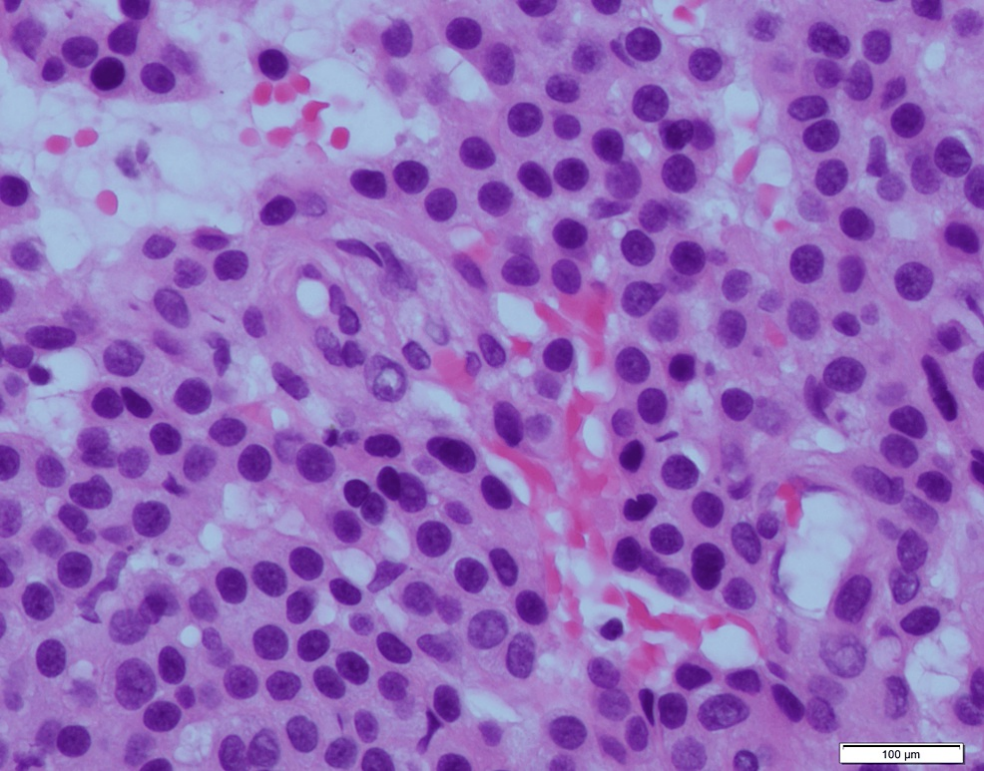
Deltoid mass biopsy at 40× magnification with H&E staining shows round, sharply demarcated cytoplasm with monotonous round nuclei, consistent with a diagnosis of glomus tumor. Not pictured: Immunohistochemical staining of the mass showed diffuse positivity for SMA and negativity for CD56, chromogranin, and synaptophysin. H&E: hematoxylin and eosin; SMA: smooth muscle actin

General surgery excised the area of interest and sent the tissue to pathology for further diagnosis. The case was eventually referred to Cleveland Clinic for outside consultation. Grossly, the specimen was a well-defined soft tissue nodule measuring 0.7×0.6×0.5 cm found on the left lateral deltoid (Figure 3). Histologically, the sections showed a well-circumscribed neoplasm consisting of proliferative epithelioid cells with centrally placed round nuclei, eosinophilic cytoplasm, and well-defined cell borders along with irregularly thin-walled blood vessels (Figure 4). Immunohistochemistry (IHC) was negative for CD56, chromogranin, and synaptophysin and was diffusely positive for smooth muscle actin (SMA). The final pathologic diagnosis was a glomus tumor. Following the surgery, the patient reported complete resolution of his left upper extremity symptoms.

## Discussion

Glomus tumors usually contain a combination of glomus cells, smooth muscle cells, and vasculature [3]. The most common variant is the solid glomus tumor, which consists of minimal vasculature and muscular components [3]. Other variants include glomangiomas, which contain prominent vascular components, and glomangiomyomas, which have prominent vascular and smooth muscle components.

Histopathologically, glomus tumors consist of branching vessels lined by endothelium and glomus cells surrounding them in a nest-like pattern [3]. Due to the focal hyalinization of small vessel walls, the vasculature usually stands out prominently on microscopy [5]. Morphologically, glomus cells are uniform with sharply defined cytoplasm and round, regular nuclei with minimal mitotic activity, making their potential for malignancy very low [5]. IHC is used for the definitive diagnosis of glomus tumors, which are known to stain positively for SMA and muscle-specific actin and stain negatively for desmin and S100 [5]. Staining for S100 is helpful in ruling out other conditions that have similar histologic presentations to glomus tumors such as melanocytic nevus [4].

While some glomus tumors can be asymptomatic, others can cause immense, focal pain through the entrapment of myelinated nerve fibers between the nests of tumor cells [3]. In a retrospective study done by Martínez-Villén et al. that looked at 541 surgeries for nerve compression due to non-neural tumors of the forearm and hand, two cases were found to be glomus tumors located on the distal forearm [6]. The study showed that glomus tumors were more likely to affect superficial nerve branches such as the superficial radial branch and dorsal sensory ulnar branch, creating a strong pain response localized to the nerve distribution, which could present clinically as sharp point tenderness [6]. The thermoregulation properties seen in normal glomus cells can also manifest clinically as paresthesias, cold intolerance, and a burning sensation as seen in this patient, making it important to consider glomus tumors in the diagnostic workup of soft tissue point tenderness [7].

Glomus tumors with non-classical presentations are commonly misdiagnosed which can lead to delays in treatment and prolongation of patient suffering. Imaging should include initial color Doppler ultrasonography potentially followed by MRI [7]. Doppler ultrasonography can detect tumors as small as 2 mm in size and provides visualization of blood flow within the tumor, allowing distinction between vascular tumors such as the glomus tumor and solid tumors [8]. However, the vascular signals of glomus tumors seen on Doppler imaging are very similar to the signals expressed by other vascular neoplasms such as hemangiomas and AV malformations [8]. Glomus tumors and vascular tumors can be further differentiated on Doppler ultrasound through manual compression [7]. Compression of a vascular tumor shows decreased blood flow with return of the original flow after cessation of compression [7]. In a glomus tumor, compression does not affect blood flow and causes significant pain [7]. B-mode, or gray-scale, ultrasound can also be used to differentiate AV malformations and hemangiomas from glomus tumors [8]. Hemangiomas show low vascular flow and blood pools, and AV malformations are poorly defined without mass effect, which differs from the sharply defined, homogenous appearance of the glomus tumor on B-mode ultrasound [8]. In the case of soft tissue point tenderness, duplex ultrasound which consists of both B-mode and Doppler should be considered for initial imaging. MRI should be used in cases where the tumor is smaller than 2 mm, Doppler ultrasound is unclear, or malignancy must be ruled out [7]. Imaging is useful for the visualization of these tumors, but IHC is required for definitive diagnosis [7]. The mainstay of treatment is surgical excision. Most patients report full resolution of symptoms and recurrence risk is low [7]. Although malignancy potential is very low in glomus tumors, excision with expanded margins should be considered with recurrent tumors or high suspicion of malignancy [7]. 

## Conclusions

Although glomus tumors rarely present on the proximal upper extremity, this case exemplifies how significantly they can affect quality of life. This patient experienced worsening chronic pain for over three decades, making an important statement that glomus tumors should be considered in the differential diagnosis of soft tissue point tenderness of the extremities. Duplex ultrasound is the most effective initial test to determine appearance and vascularity, but imaging must be clinically correlated with patient presentation and physical exam findings to avoid misdiagnosis. Management with excision is curative; if clinical suspicion for a glomus tumor is high, excision should be done promptly. IHC must be done for definitive diagnosis and to rule out malignancy. Due to limited information on non-classical glomus tumors, this report will aid in furthering knowledge of clinical presentation, diagnostic workup, and treatment.
